# Oxidation Resistance, Ablation Resistance, and Ablation Mechanism of HfC–B_4_C-Modified Carbon Fiber/Boron Phenolic Resin Ceramizable Composites

**DOI:** 10.3390/polym17101412

**Published:** 2025-05-20

**Authors:** Hairun Wen, Wei Zhang, Zongyi Deng, Xueyuan Yang, Wenchao Huang

**Affiliations:** 1 School of Materials Science and Engineering, Wuhan University of Technology, Wuhan 430070, China; 331418@whut.edu.cn (H.W.); wezhang@whut.edu.cn (W.Z.); zong_yi_deng@whut.edu.cn (Z.D.); 2 Hubei Longzhong Laboratory, Wuhan University of Technology Xiangyang Demonstration Zone, Xiangyang 441000, China

**Keywords:** HfC, B_4_C, ceramizable composite, oxidative corrosion, flexural strength, ablation mechanism

## Abstract

Thermal protection materials with excellent performance are critical for hypersonic vehicles. Carbon fiber/phenolic resin composites (C_f_/Ph) have been widely used as thermal protection materials due to their high specific strength and ease of processing. However, oxidative failure limits the extensive applications of C_f_/Ph in harsh environments. In this paper, a novel hafnium carbide (HfC) and boron carbide (B_4_C)-modified C_f_/Ph was fabricated via an impregnating and compression molding route. The synergistic effect of HfC and B_4_C on the thermal stability, flexural strength, microstructure, and phase evolution of the ceramizable composite was studied. The resulting ceramizable composites exhibited excellent resistance to oxidative corrosion and ablation behavior. The residual yield at 1400 °C and the flexural strength after heat treatment at 1600 °C for 20 min were 46% and 54.65 MPa, respectively, with an increase of 79.59% in flexural strength compared to that of the composites without ceramizable fillers. The linear ablation rate (LAR) and mass ablation rate (MAR) under a heat flux density of 4.2 MW/m^2^ for the 20 s were as low as −8.33 × 10^−3^ mm/s and 3.08 × 10^−2^ g/s. The ablation mechanism was further revealed. A dense B–C–N–O–Hf ceramic layer was constructed in situ as an efficient thermal protection barrier, significantly reducing the corrosion of the carbon fibers.

## 1. Introduction

In recent years, with the rapid development of aerospace technology, the flight speed of hypersonic vehicles is continuously increasing and the service environment is becoming increasingly harsh, leading to significant damage to the vehicle’s material structure [1,2,3,4]. An effective thermal protection system (TPS) would provide adequate protection for the hypersonic vehicle structure against intense aerodynamic heating, allowing the vehicle to remain safe during the flight period. Thermal protection materials, which are key factors in the TPS, can isolate the high-temperature environment and prevent flame penetration by absorbing substantial heat energy through various physical and chemical processes, including decomposition, melting, evaporation and sublimation, as well as by generating pyrolysis gases within the material under severe heat flux, thereby ensuring the structural integrity and normal operation of the hypersonic vehicles [5,6,7]. Therefore, it is crucial to develop TPMs with superior performance. Carbon fiber/phenolic resin composites (C_f_/Ph) are widely used in ablative materials due to their advantages of lightweight and high strength, easy molding process and the possibility of mass molding. However, at ultra-high temperatures, carbon fibers and phenolic resin undergo rapid pyrolytic and oxidative degradation, compromising the residual strength and structural integrity of the composites and resulting in diminished material properties. Therefore, ordinary carbon fiber-reinforced phenolic resin composites can no longer meet the thermal protection demands at elevated temperatures, and it is necessary to improve their high-temperature ablative and oxidative resistance.

Ultra-high-temperature ceramics (UHTCs), such as ZrC [8,9], hafnium carbide (HfC) [10], TiC [11,12], ZrB_2_ [13], HfB_2_ [14], TiB_2_ [15], and SiC [16], exhibit high thermal stability and superior mechanical properties, including high hardness, high mechanical strength, and excellent abrasion resistance, enabling them to withstand extreme operating conditions characterized by high temperatures and high mechanical loads. In recent years, UHTCs have been continuously and extensively applied and studied in the field of ceramizable phenolic resin composites. For example, Shi et al. [17] modified aluminized carbon fiber/boron phenolic composites with TiB_2_ and B_4_C, resulting in a residual yield of 90.4% and a flexural strength of 53.1 MPa after a 15 min heat treatment at 1400 °C. The residual yield was increased by 15.9%, and the flexural strength was increased by 532.1% compared to that of the material with no sinterable filler added. Zou et al. [18] modified C_f_/Ph using the HfB_2_–SiB_6_ system, and the experimental results showed that the composites achieved a ceramic yield of 69.69% at 1600 °C, and their linear ablation rate (LAR) and mass ablation rate (MAR) were as low as 0.004 mm/s and 0.043 g/s, respectively, for 20 s at a heat flux density of 4.2 MW/m^2^. Deng et al. [19] investigated the effect of LaB_6_-modified Cf/Ph in terms of ablation behavior, microstructure, phase evolution, and in situ ceramization mechanism. The results showed that the residual yield of the material reached 32.8% at 1500 °C and that the material formed dense B–C–O–Zr–La ceramic layers in situ at high temperatures, and these layers had a mosaic structure, which significantly reduced the corrosion of carbon.

Boron carbide (B_4_C) is usually employed as a sintering aid since it forms a molten phase at elevated temperatures, which could fill and heal cracks from resin pyrolysis and penetrate oxygen further diffusing [15,20,21,22,23]. In this work, we fabricated novel HfC and B_4_C synergistically modified carbon fiber/boron phenolic resin ceramizable composites via an impregnating and compression molding route. The thermal stability and mechanical properties of the materials with different contents of B_4_C were studied, and the mechanism of oxidation resistance and ablative resistance at high temperatures is discussed.

## 2. Materials and Methods

### 2.1. Raw Materials

Carbon fiber scrim (W-7022FF-400) with a surface density of 400 gsm, purchased from Weihai Guangwei Composites Co. (Weihai, China), was used as reinforcement. Thermosetting boron phenolic resin (BPR, THC-400) with 7 wt.% boron content, provided by Shaanxi Taihang Refractory Polymers Co. (Weinan, China), was used as the matrix. HfC powder (99.9%), with an average particle size of 1–10 µm, was provided by Shanghai Chaowei Nanoscience Co. (Shanghai, China). B_4_C powder (98%) with an average particle size of 1-10 µm was purchased from Shanghai Macklin Biochemical Technology Co. (Shanghai, China), Ltd. The HfC powder and B_4_C powder were used together as ceramizable fillers. Anhydrous ethanol (AR), purchased from China Pharmaceutical Group Chemical Reagent Co. (Shanghai, China), was used as the solvent. All raw materials were used directly without further purification.

### 2.2. Fabrication of Ceramizable Composites

**Figure 1:**
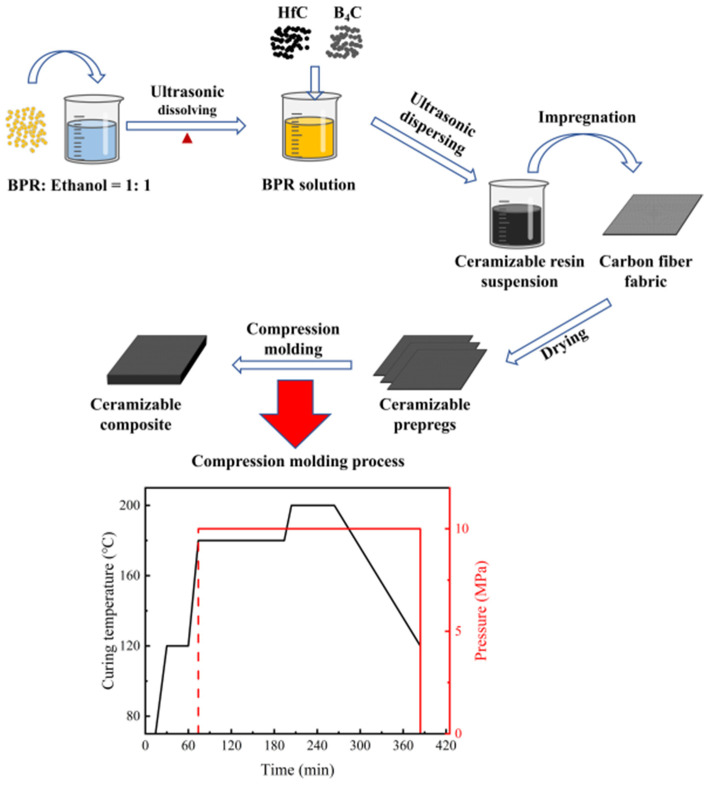
Schematic diagram of the preparation process and curing process for ceramizable composites.

The ceramizable composites were fabricated via impregnation and compression molding, as depicted in Figure 1. First, BPR was mixed with anhydrous ethanol in a 1:1 mass ratio under constant magnetic stirring at 600 rpm for 30 min. The yellow BPR solution was obtained by ultrasonicating the mixture at 80 °C for 30 min. Second, the ceramizable filler, as specified in Table 1, was added to the BPR solution and magnetically stirred for 10 min, followed by ultrasonic dispersion for an additional 10 min to achieve a well-dispersed suspension. Third, the carbon fiber scrim fabric was uniformly coated with the ceramizable BPR solution to ensure complete impregnation. The impregnated fabric was air-dried at room temperature to obtain the ceramizable prepreg. The prepregs are then stacked, molded, and cured using a compression process. In this case, 23 layers of prepreg were stacked for samples used for ablative testing and 9 layers of prepreg were stacked for samples used for heat treatment and flexural testing.

### 2.3. Flexural Test

The flexural strengths of the experimentally prepared composites before and after heat treatment were measured using a universal mechanical testing machine (RGM-4100, Ruge Instrument Co., Ltd., Shenzhen, China). The heat treatment procedure was as follows: Samples were placed in a high-temperature chamber furnace (KSL-1700, Kejing Co., Shenzhen, China) for 20 min at temperatures of 600 °C, 800 °C, 1000 °C, 1200 °C, 1400 °C, and 1600 °C, respectively. After heat treatments, the samples were removed and their flexural strengths were assessed using the three-point flexural method, following the guidelines of the Chinese standard GB/T 1449-2005 [24]. The test parameters included a span of 16d and a crosshead speed of 2 mm/min.

(1) $$\sigma =3Fl/\left(2bd^{2}\right)$$

where σ is the flexural strength of the sample, F is the maximum load that the sample is subjected to, b is the width of the sample, and d is the thickness of the sample. The final results were taken as the arithmetic mean of four parallel tests.

### 2.4. Oxy-Acetylene Ablation Test

The test was conducted according to the oxy-acetylene flame test method stipulated in the national military standard GJB 323A-1996 [25]. Among standard, the gas flow rate of oxygen was 1512 L/h, the gas pressure was 0.4 MPa, the gas flow rate of acetylene was 1116 L/h, the gas pressure was 0.095 MPa, the mixing ratio of oxygen and acetylene was 1.35, the heat flow density of the flame was 4.2 ± 0.2 MW/m^2^, the flame temperature reached as high as 3000 °C [26,27], the nozzle diameter was 2 mm, the vertical distance between the muzzle and the surface of the composite material was 10 mm, and the ablation time Δt = 20s. The line ablation rate and mass ablation rate were calculated based on the depth change and mass change.

(2) $$LAR=\left(d_{1}-d_{2}\right)/∆t$$

(3) $$MAR=\left(m_{1}-m_{2}\right)/∆t$$

where d_1_ and d_2_ are the initial thickness of the sample and the thickness after the ablation test, respectively, and m_1_ and m_2_ are the initial mass of the sample and the mass after the ablation test. The final results were taken as the arithmetic mean of five parallel tests.

### 2.5. Characterizations

The microstructure of the ceramizable composites was observed by using a ZEISS SIGMA 300 (Carl Zeiss AG., Jena, Germany) scanning electron microscope (SEM) with an accelerating voltage of 15 keV. Additionally, the elemental distribution was analyzed using an Oxford energy-dispersive X-ray (EDS) spectrometer (Carl Zeiss AG., Jena, Germany). Thermal stability and oxidative corrosion resistance were assessed by using a NETZSCH STA 449 F3 Jupiter simultaneous thermal analyzer (Naichi Scientific Instrument Trading Co., Ltd., Selb, Germany) in the air at a heating rate of 10 °C/min over a temperature range of 0–1400 °C. Residual yield was calculated from Equation (4).

(4) $$R=m_{4}/m_{3}$$

where R is the residual yield, and m_3_ and m_4_ are the mass of the sample before and after the thermogravimetric tests, respectively.

The Gibbs free energy change was calculated from Equation (5).

(5) ∆G=∆H−T∆S

where ΔG is the Gibbs free energy change, ΔH is the enthalpy change in the chemical reaction, ΔS is the entropy change, and T is the temperature. ΔG was estimated and provided by the HSC Chemistry 6.0 software package.

The crystal phases were characterized by using X-ray diffraction (XRD) with a D8 Advance model (Bruker Co., Berlin, Germany). Testing parameters were in the range of 5–80°, a scanning rate of 4°/min, a test voltage of 40 kV, and a test current of 400 mA. The crystallinity of the samples was calculated using the split peak fitting method. The lattice constants of the crystals and the mass shares of the components were estimated by MDI Jade 6.5. Elemental compositions were determined using a Thermo Scientific ESCALAB 250Xi X-ray photoelectron spectrometer (XPS) (Thermo Fisher Scientific Co., Waltham, MA, USA). The pass energy and step size for the survey scan were set at 100 eV and 1.0 eV, respectively, while for high-resolution scans were 30 eV and 0.1 eV, respectively.

## 3. Results and Discussion

### 3.1. Density and Porosity of the Ceramizable Composites

**Figure 2:**
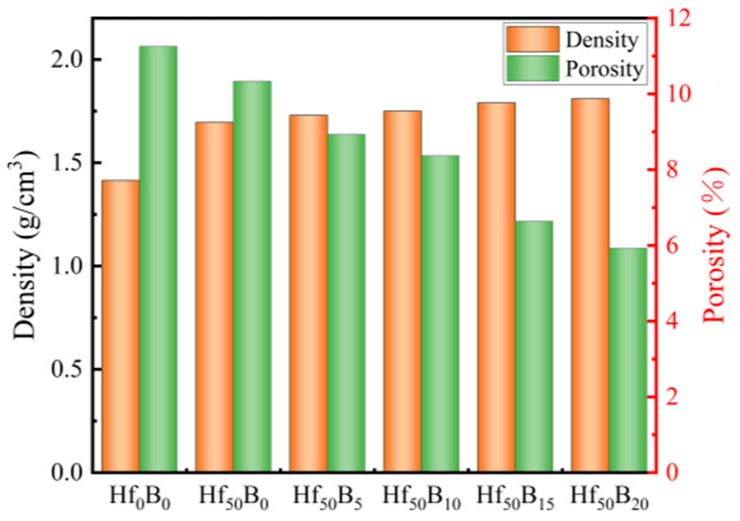
Density and porosity of ceramizable composites.

During the heating phase of sample preparation, low-boiling-point substances such as formaldehyde and phenol dissolved in the resin will vaporize, and if they are not discharged promptly, the flowing resin will be extruded during the pressurization phase, resulting in a decrease in the density of the sample. In addition, small molecule gases are released during the curing and cross-linking stages to form pores within the material, affecting the denseness of the material. Figure 2 illustrates the density and porosity of the different formulations of the ceramizable composites. The results showed that the addition of both HfC and B_4_C decreased the porosity of the material. This is due to the fact that during the curing cross-linking stage, HfC and B_4_C occupied the position of pores and improves the densification of the composites. In addition, the viscosity of the resin fluid increased and less mass was extruded during pressurization. As a result, the density of the ceramizable composites increased from 1.414 g/cm^3^ for Hf_0_B_0_ to 1.695 g/cm^3^ for Hf_50_B_0_ and then gradually increased to 1.810 g/cm^3^ for Hf_50_B_20_. It is noteworthy that the density of the ceramizable composites was less compared to that of the C/C and ceramic-based composites of similar systems whose densities have been reported [28,29]. This indicates that the ceramizable composites have the advantage of being lightweight.

### 3.2. Thermal Stability, Oxidation Resistance, and Flexural Strength of Ceramizable Composites

**Figure 3:**
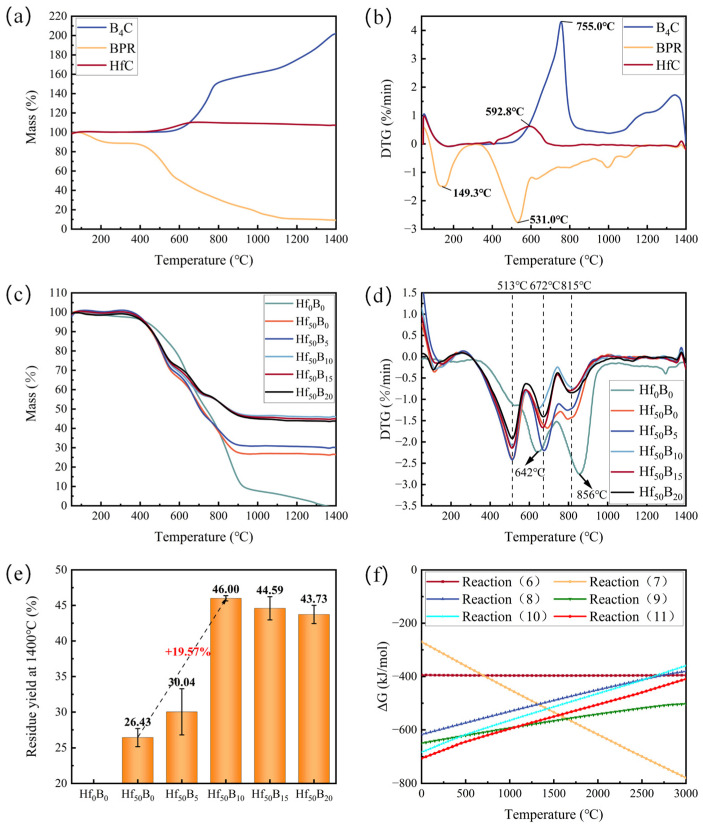
(**a**) TGA curves, (**b**) DTG curves for single components, (**c**) TGA curves, (**d**) DTG curves for composites, (**e**) residuals yield at 1400 °C, and (**f**) ΔG curves for oxidation reactions of reactions (6)–(11).

Figure 3 illustrates the thermal stability of the materials in air. Figure 3a,b show the thermogravimetric analysis (TGA) as well as the derivative thermogravimetry (DTG) curves of each single component material in air. It can be seen that the mass of BPR was in a decreasing trend, in which the peak decomposition rate temperature of small molecule gasification was 149.3 °C. The peak decomposition rate temperature for the violent cracking reaction was 531.0 °C. The pyrolysis process of BPR has been previously reported. The primary pyrolysis products between 350 °C and 850 °C include phenols, toluene, styrene, aldehydes, carboxylic acids, CO, CO_2_, H_2_O, and long-chain olefins such as squalene [23]. Additionally, the main chain of BPR undergoes scission, forming polycyclic aromatic hydrocarbon structures. These structures will subsequently undergo further carbonization to form pyrolytic carbon (PyC). HfC and B_4_C were involved in the reactions (8)–(11) to increase the residuals through the fixation of oxygen. HfC began to be oxidized at about 400 °C with a maximum reaction rate temperature of 592.8 °C. B_4_C began to be oxidized at about 500 °C with a maximum reaction rate temperature of 755.0 °C.

(6) $$C+O_{2}(g)=CO_{2}(g)$$

(7) $$2C+O_{2}(g)=2CO(g)$$

(8) $$1/2HfC+O_{2}(g)=1/2HfO_{2}+1/2CO_{2}(g)$$

(9) $$2/3HfC+O_{2}(g)=2/3HfO_{2}+2/3CO(g)$$

(10) $${1/4B}_{4}C+O_{2}(g)=1/2B_{2}O_{3}+1/4CO_{2}(g)$$

(11) $${2/7B}_{4}C+O_{2}(g)=4/7B_{2}O_{3}+2/7CO(g)$$

Figure 3c shows the TGA curves of the ceramizable composites in the air. The results showed that the temperature for 0% residual yield of Hf_0_B_0_ was 1347 °C. The addition of HfC resulted in a 26.43% residual yield of Hf_50_B_0_. The addition of B_4_C significantly increased the mass retention rate at high temperatures, with Hf_50_B_10_ having the highest residual yield of 46.00%, which increased by 19.57% compared with that of Hf_50_B_0_ (Figure 3e). When the content of B_4_C exceeded 10 phr, the residual yield of the composites began to decrease. This is attributed to the relatively high saturated vapor pressure of B_2_O_3_ at elevated temperatures, which facilitates its volatilization [19].

Figure 3d shows the DTG curves of the composites. The results showed that the peak decomposition temperatures of Hf_0_B_0_ were 642 °C and 856 °C. After the addition of HfC, the peak decomposition temperatures of Hf_50_B_0_ increased to three. Among them, the first peak decomposition temperature was advanced to 513 °C and the second peak temperature was advanced to 672 °C. The possible reason for the leftward shift in the peak decomposition temperature is that the addition of HfC increased the thermal conductivity of the composite, and heat was thus conducted faster, leading to the earlier cracking of the BPR. With the continued addition of B_4_C, the thermal conductivity of the composite did not increase significantly, so the peak decomposition temperature remained essentially unchanged. The thermal conductivity of the composites is shown in Table S1. Between 513 °C and 672 °C, the oxidation processes of HfC and B_4_C offset the mass loss from resin cracking and carbon oxidation, while B_4_C continued to participate in the oxidation reaction between 672 °C and 815 °C. The generated B_2_O_3_, with a low melting point (~450 °C), melts and flows at high temperatures and fills defects such as pores generated by the pyrolysis process, reducing the O_2_ entry into the interior of the material to contact and react with the carbon fiber pathway [5,14]. As a result, the decomposition degree of the material gradually decreases above 815 °C.

Figure 3f illustrates the Gibbs free energy change curves of carbon fiber, HfC, and B_4_C involved in the oxidation reaction. The results show that at temperatures less than 1300 °C, HfC and B_4_C ceramics have lower ΔG values for the oxidation reaction and therefore react more easily with O_2_. During the pyrolysis process, HfC and B_4_C participated in oxidation reactions, consuming oxygen and simultaneously generating a substantial amount of CO and CO_2_ gases. These gases reduced the oxygen concentration at the material surface, thereby mitigating the pyrolysis of the resin and the oxidative corrosion of the carbon fibers.

**Figure 4:**
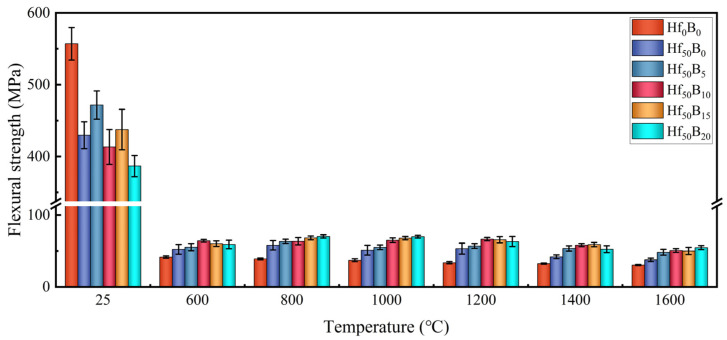
Flexural strength of ceramizable composites before and after heat treatment at various temperatures.

Figure 4 shows the flexural strength of the ceramizable composites before and after heat treatment at various temperatures. It can be seen that before heat treatment, the addition of ceramics gave an overall decreasing trend in the flexural strength of the composites. This is because the increase in content of ceramizable filler hindered the bonding of the resin with the carbon fibers, and the interfacial bonding force was subsequently reduced. Microcracks were more likely to occur within the composite material during the flexural test, which was macroscopically reflected in the reduction in the flexural strength. As a result of heat treatment, the flexural strength of the material decreased in a cliff-like manner. This was due to the weakening of the interlayer bonding during high-temperature oxidative corrosion and the generation of pores and microcracks within the composite. When bearing loads, the pores and microcracks were more likely to expand until the material broke.

Comparing the flexural strengths of Hf_0_B_0_ and Hf_50_B_0_, it can be seen that the addition of HfC significantly improved the flexural strength of the composites after heat treatment. Firstly, HfC was easier to oxidize and produced CO_2_ gas, which created an oxygen-deficient atmosphere local. Secondly, after heat treatment, the dispersed HfC and HfO_2_ particles produced a “pinning effect”, preventing cracks from spreading, and thus the flexural strength was significantly improved.

The addition of B_4_C could help to fill and heal the generated pores and cracks during the resin pyrolysis through the flow of molten B_2_O_3_ at high temperatures. With the increasing content of B_4_C, more pores and cracks would be healed. As a result, the flexural strength of the heat-treated composites rose overall with increasing B_4_C content. Between 800 °C and 1200 °C, the flexural strength of Hf_50_B_10_, Hf_50_B_15_, and Hf_50_B_20_ did not change much. This is because in this temperature interval, B_4_C, with the highest oxidation rate, preferentially participated in the oxidation process. When the heat treatment temperature reached 1400 °C, most B_2_O_3_ was vaporized, the filled pores and cracks were re-exposed, and the surface and interior of the material were subjected to more severe high-temperature oxidative corrosion, and the flexural strength showed a significant decrease. After the temperature reached 1600 °C, the change in flexural strength was not as large as that at 1400 °C, which was attributed to the decrease in the cross-sectional area of the strip samples after heat treatment at 1600 °C. As a result, the synergistic effect of HfC and B_4_C resulted in the flexural strength of Hf_50_B_20_ of 54.65 MPa after heat treatment at 1600 °C, which increased by 79.59% and 44.84% by comparing with that of Hf_0_B_0_ and Hf_50_B_0_, respectively. Compared with the flexural strength results of similar systems, the advantages of the HfC-B_4_C system are evident (Figure S1 and Table S2).

### 3.3. Ablation Resistance of Ceramizable Composites

**Figure 5:**
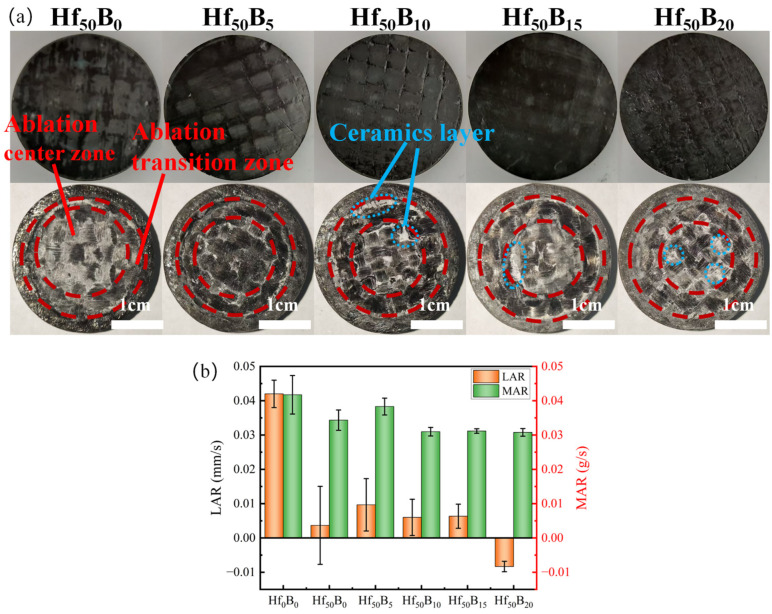
(**a**) Macroscopic morphology before and after oxy-acetylene ablation, and (**b**) ablation rates.

In the oxy-acetylene ablation test, samples were exposed to an oxy-acetylene flame with a heat flux density of 4.2 MW/m^2^ for 20 s. The enhancements of ablation resistance by HfC and B_4_C were investigated. Figure 5a illustrates the surface images of the samples before and after ablation, where the surface of the samples is divided into three areas by dashed lines: the ablation center zone, the ablation transition zone, and the edge zone. The image shows that the ablation area in the center decreases with the increase in the B_4_C content. Between the intervals of the areas of Hf_50_B_10_, Hf_50_B_15_, and Hf_50_B_20_, there are white ceramic layers, which are the result of the combination of high-speed airflow scouring and B_2_O_3_ glass phase adhesion.

Figure 5b depicts the linear and mass ablation rates of the ceramizable composites. The LAR and MAR of Hf_0_B_0_ were 4.20 × 10^−2^ mm/s and 4.17 × 10^−2^ g/s, respectively. The LAR and MAR of Hf_50_B_0_ with the addition of HfC were 3.67 × 10^−3^ mm/s and 3.43 × 10^−2^ g/s, respectively. The results showed that the LAR was reduced by an order of magnitude, and that the incorporation of HfC enhanced the material resistance to ablation by an extremely high degree. Under the flame of oxy-acetylene at 3000 °C, B_2_O_3_ evaporates or sublimates rapidly, and part of the HfO_2_ (melting point 2758 °C) also melts and sticks to the carbon fiber. HfC (melting point 3958 °C), which has a very high melting point, is resistant to high-temperature ablation. The increased mass ablation rate for Hf_50_B_5_ compared to Hf_50_B_0_ is due to the volatilization of B_2_O_3_, whereas the reduced mass ablation rates for Hf_50_B_10_, Hf_50_B_15_, and Hf_50_B_20_ were due to the fact that the ceramic particles and cracked carbon were adhered by the B_2_O_3_ glassy phase, which reduces the amount of mass that could be stripped off by the high-velocity gas stream. Notably, the LAR and MAR of Hf_50_B_20_ were −8.33 × 10^−3^ mm/s and 3.08 × 10^−2^ g/s, respectively, with a 9.9% decrease in MAR compared to Hf_50_B_0_. The transition of the LAR from positive to negative indicates that the volume of the composite expanded during the ablation process, and the increase in thickness prolonged the ablation resistance time. It was expected that the composites would have a longer operational time in harsh aerodynamic environments. The HfC–B_4_C system has a significant advantage in terms of ablation performance when compared to ablation test results from various systems with consistent ablation conditions (Figure S2 and Table S3).

### 3.4. Microstructure Evolution

**Figure 6:**
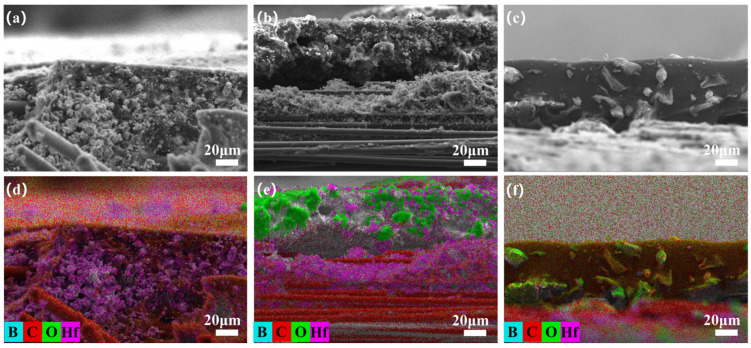
Cross-sectional microscopic images of flexural samples (**a**) Hf_50_B_0_, (**b**) Hf_50_B_10_, and (**c**) Hf_50_B_20_, as well as EDS mapping of corresponding samples (**d**) Hf_50_B_0_, (**e**) Hf_50_B_10_, and (**f**) Hf_50_B_20_.

To deeply investigate the effect of ceramizable fillers on the oxidation resistance of the composites, we observed the cross-sectional microstructure of the oxide layer on the surface of the flexural samples and recorded elemental mappings to better analyze the elemental distribution. During heat treatment, the resin cracked to produce gases, leading to a decrease in interfacial strength. Concurrently, the escape of these gases increased interlayer stresses, which disrupted the structure of the material and created numerous pores, leading to delamination and peeling-off under severe conditions. Figure 6a shows a cross-sectional micrograph of Hf_50_B_0_ after heat treatment at 1600 °C for 20 min. An important consequence of the oxidation of HfC was the formation of a fluffy, porous, coral-like HfO_2_ structure (Figure 6d), where stresses generated by volumetric expansion during the crystallization process led to more pores, acting as conduits for the continuous inward erosion of O_2_ and heat flow. After adding 10 phr of B_4_C, the oxide layer on the surface of the composite material melted and flowed to form a ceramic layer (Figure 6b,e), which exhibited a repairing effect on some of the pores. However, the melt was not enough, and the ceramic layer on the surface was loose and not able to tightly bond with the interior, with cracks still present. When the B_4_C content was increased to 20 phr, a ceramic layer with uniform thickness and close bonding between the inside and outside was formed on the surface of the composite material (Figure 6c,f), and the pores were filled by B_2_O_3_ melt. This not only reduced the pathways for oxidation and heat flow corrosion but also bonded the HfO_2_ particles and PyC together, resulting in a denser structure.

**Figure 7:**
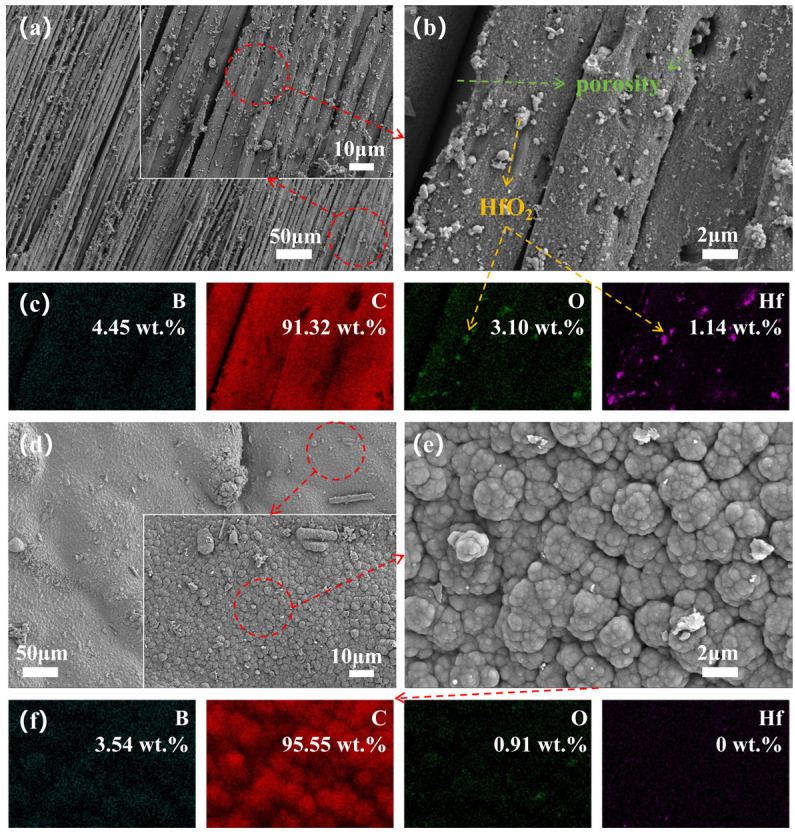
(**a**,**b**) Microscopic images of the ablation center of Hf_50_B_0_, and (**c**) EDS mapping of (**b**); (**d**,**e**) microscopic images of the ablation transition zone of Hf_50_B_0_, and (**f**) EDS mapping of (**e**).

To elucidate the significant differences in ablation behavior, we observed the surface micro-morphology of the central region and transition zone of the ablated surface and recorded elemental mappings for a detailed analysis of elemental distribution. The microscopic images and elemental distribution of the ablated surface for Hf_50_B_0_ are depicted in Figure 7. The ablation center exhibits a multitude of exposed carbon fibers along with minor PyC and ceramic particles, suggesting severe ablation damage to Hf_50_B_0_. The bonding of HfC, HfO_2_, and PyC with the resin and carbon fibers was very fragile and easily detached under the washing effect of the high-speed heat flow. Conversely, in the ablation transition zone, extensive PyC formation occurred without exposed carbon fibers, attributed to the diminished temperature and scouring effects at the flame’s periphery.

**Figure 8:**
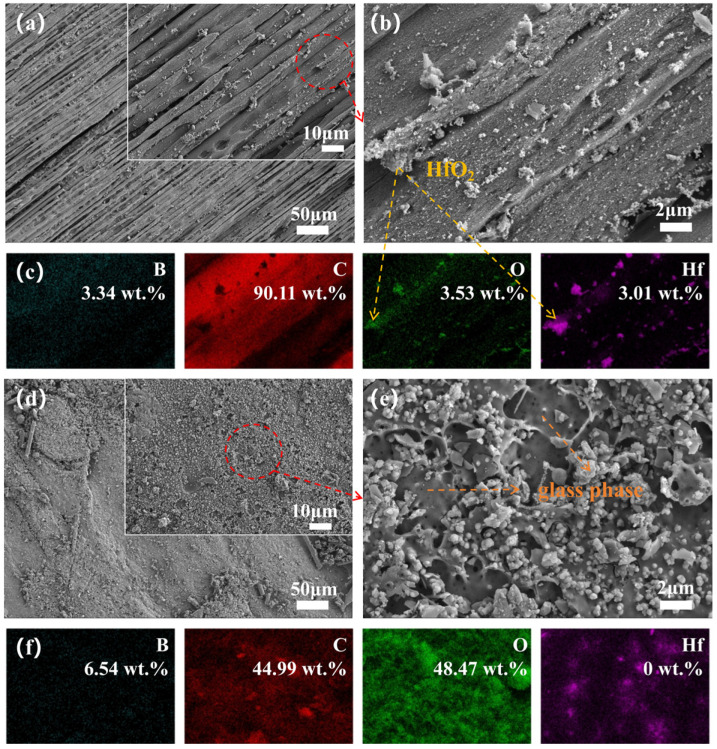
(**a**,**b**) Microscopic images of the ablation center of Hf_50_B_5_, and (**c**) EDS mapping of (**b**); (**d**,**e**) microscopic images of the ablation transition zone of Hf_50_B_5_, and (**f**) EDS mapping of (**e**).

The morphology of the ablation centers in Hf_50_B_5_ (Figure 8a) appears to be no better than that in Hf_50_B_0_. Surrounding the Hf element, an oxide distinct from HfO_2_ (Figure 8b,c) was observed, identified as B_2_O_3_, suggesting that only a small amount of molten B_2_O_3_ acted as an adhesive in the high-temperature environment. This is partly due to the low content of B_4_C and, on the other hand, the high-temperature volatilization and the scouring of the high-speed heat flow reduced the content of B_2_O_3_ on the surface of the material. In contrast to Hf_50_B_0_, the ablation transition zone of Hf_50_B_5_ is a scene of oxides mixed with PyC, and most of these oxides are B_2_O_3_ glass phase.

**Figure 9:**
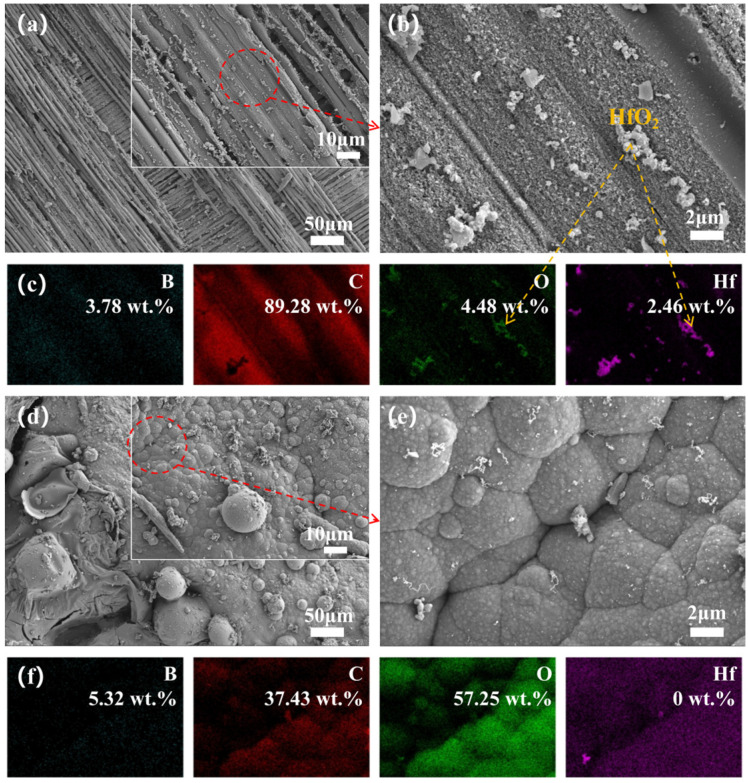
(**a**,**b**) Microscopic images of the ablation center of Hf_50_B_10_, and (**c**) EDS mapping of (**b**); (**d**,**e**) microscopic images of the ablation transition zone of Hf_50_B_10_, and (**f**) EDS mapping of (**e**).

Hf_50_B_10_ exhibits continuous carbon layers at the center of the ablated surface (Figure 9b), originating from boron phenolic resin. The area of exposed carbon fibers was reduced, but relative to the presence of multiple broken carbon fibers still can be seen, indicating that the oxidative corrosion of the carbon fibers and PyC was still severe. In the ablation transition zone, the mass percentage of Hf was 0 wt.%. There are two possible reasons. One is that the oxides and PyC generated on the surface covered the Hf-based material. The other is that the Hf material was washed away from the surface by the high-speed heat flow. The high-temperature environment in the ablation transition zone resembles static calcination, and considering the morphology of the static calcination cross-section (Figure 6b), the first scenario appears more plausible.

**Figure 10:**
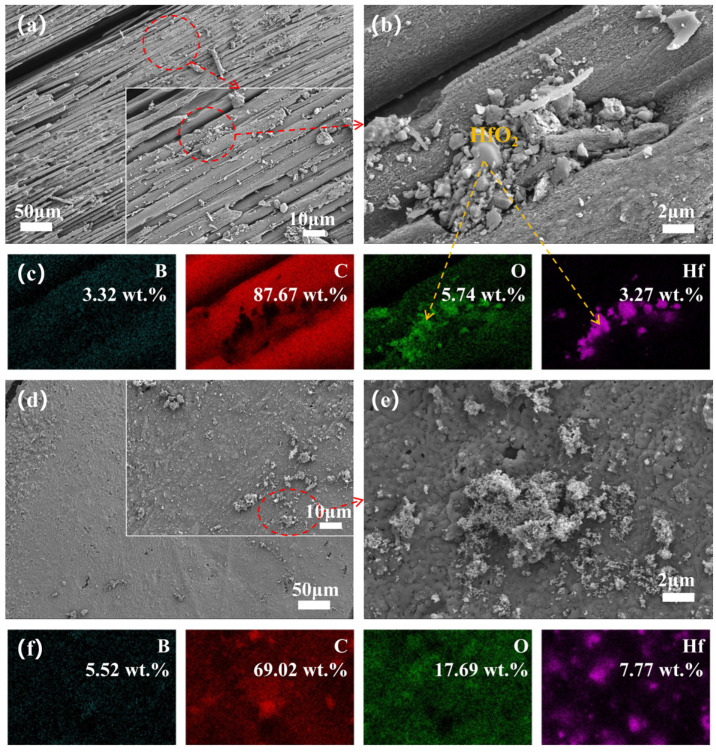
(**a**,**b**) Microscopic images of the ablation center of Hf_50_B_15_, and (**c**) EDS mapping of (**b**); (**d**,**e**) microscopic images of the ablation transition zone of Hf_50_B_15_, and (**f**) EDS mapping of (**e**).

**Figure 11:**
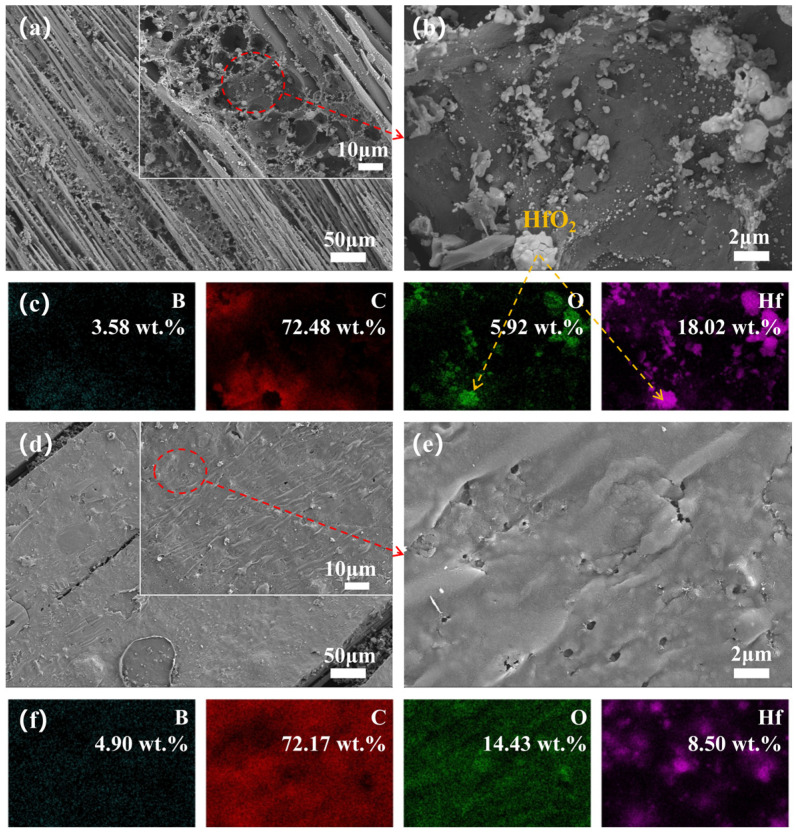
(**a**,**b**) Microscopic images of the ablation center of Hf_50_B_20_, and (**c**) EDS mapping of (**b**); (**d**,**e**) microscopic images of the ablation transition zone of Hf_50_B_20_, and (**f**) EDS mapping of (**e**).

The ablation centers of Hf_50_B_15_ (Figure 10a) and Hf_50_B_20_ (Figure 11a) were similar to those of Hf_50_B_10_, but the surfaces were covered with more PyC, which suggests that oxidative corrosion was further reduced. In the ablation transition zone, both Hf_50_B_15_ (Figure 10e) and Hf_50_B_20_ (Figure 11e) formed a ceramic layer with a flat surface. Among them, Hf_50_B_20_ had fewer holes on the surface and the ceramic layer was denser.

### 3.5. Phase Evolution

**Figure 12:**
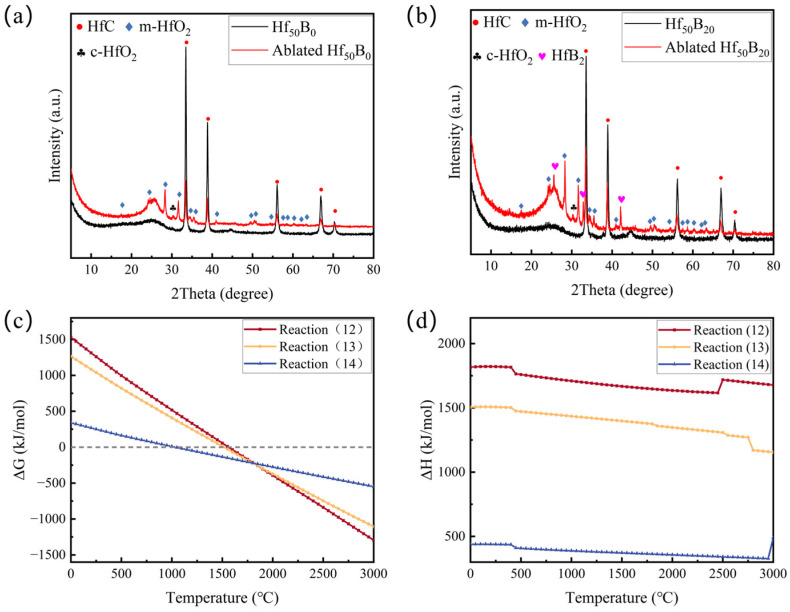
XRD images of (**a**) Hf_50_B_0_ and (**b**) Hf_50_B_20_, and (**c**) ΔG and (**d**) ΔH of the carbothermal reaction.

The phases of the composites were characterized and analyzed using XRD and XPS techniques. An observation of the XRD image of Hf_50_B_0_ (Figure 12a) reveals that the ablated sample contains HfC (PDF#39-1491), m-HfO_2_ (PDF#34-0104), and trace amounts of c-HfO_2_ (PDF#53-0560). In addition, there was a broad peak between 20° and 30°, which is characteristic of amorphous diffraction. After fitting, the crystallinity of Hf_50_B_0_ was calculated to be 69.00%. The mass ratio of HfC, m-HfO_2_, and c-HfO_2_ was 53.34: 45.92: 0.74.

(12) $$2B_{2}O_{3}+7C=B_{4}C+6CO(g)$$

(13) $$HfO_{2}+5C+B_{2}O_{3}=HfB_{2}+5CO(g)$$

(14) $$B_{2}O_{3}+3C+N_{2}(g)=2BN+3CO(g)$$

In the XRD spectrum of Hf_50_B_20_ (Figure 12b), the characteristic sharp peaks at 2θ = 25.6°, 32.8°, and 42.1° demonstrate the presence of HfB_2_ (PDF#38-1398) in the composite material, which has excellent ablation and oxidation resistance as a kind of ultra-high-temperature ceramic [30,31,32]. The crystallinity of Hf_50_B_20_ was 62.25%, and the mass ratio of HfC, m-HfO_2_, c-HfO_2_, and HfB_2_ was 29.04:62.75:1.07:7.14. More HfO_2_ adhered to the ablation surface. The area of the broad peak in Hf_50_B_20_ is much larger, indicating that more amorphous phase material was generated on the ablation surface. In the present system, the amorphous phase substances may include but are not limited to B_2_O_3_ and PyC [33,34,35]. Cell pictures and lattice parameters of all crystals were organized in Figure S3 and Table S4.

Figure 12c,d demonstrate the Gibbs free energy change curves and enthalpy change curves for reactions (12)–(14). During the ablation process, these reactions have ΔG < 0, all of which tend to react spontaneously. Moreover, these reactions have ΔH > 0, which could absorb heat during the reaction and slow down the rate of temperature rise on the material surface. The gases such as CO generated by the reactions could form a gas film as well as dilute the oxygen concentration on the surface of the material, thus reducing the oxidative corrosion of the material and the mechanical stripping caused by the high-speed airflow.

**Figure 13:**
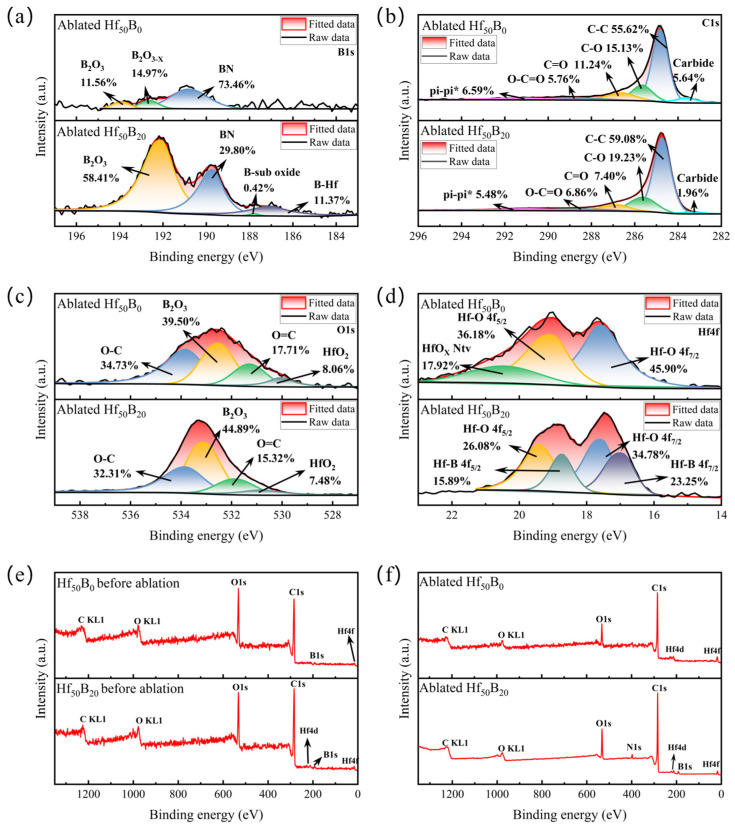
XPS narrow spectra of Hf_50_B_0_ and Hf_50_B_20_ after ablation (**a**) B1s, (**b**) C1s, (**c**) O1s, (**d**) Hf4f, and (**e**) before and (**f**) after ablation of full XPS spectra.

Figure 13a,b show the XPS narrow-spectrum images of B1s and C1s of the ablated phases of Hf_50_B_0_ and Hf_50_B_20_. The B elements in Hf_50_B_0_ are derived from BPR. The results showed that most of the B elements in Hf_50_B_0_ were involved in the reaction (14) to produce BN. The presence of BN enhances the interface bonding, leading to a more dense surface structure and improved ablation resistance [36,37,38]. However, the thermal conductivity of the material is also increased [39]. The remaining fraction forms complete B_2_O_3_ and the other fraction forms less oxygenated B_2_O_3−X_. In Hf_50_B_20_, there is also a fraction of elemental B that reacts with HfO_2_ to form HfB_2_ (reaction (13)). The binding energies of both BN and B_2_O_3_ were reduced by adding 20 phr of B_4_C. It indicates that the excessive B content leads to the increase in disorder inside the glassy phases of BN and B_2_O_3_. HfB_2_ was not detected in Hf_50_B_0_, the probable reason for this is that the formed ceramic particles are stripped by the high velocity gas flow, and thus are not able to be retained on the ablative surface. The absence of relevant characteristic peaks in XRD indicates that the BN was amorphous α-BN. No B_4_C was detected, indicating that all of the B_4_C on the surface was involved in the reaction, and also indicating that the amount of B_4_C produced by the reaction (12) was too low. XPS narrow spectra of C1s showed that the cleavage behavior of BPR did not change with the addition of B_4_C, and the cleavage products contained phenols, aldehydes, ethers, carboxylic acids, and graphite. The extent of graphitization of cracked carbon increased with an increasing B_4_C content (Figure S4). The carbide was unreacted HfC. The narrow spectrum of O1s further confirmed the cleavage behavior of BPR (Figure 13c).

Figure 13d shows the XPS narrow spectrum of Hf4f after ablation of Hf_50_B_0_ and Hf_50_B_20_. The results show that in Hf_50_B_0_, the Hf element exposed on the surface continues to react to form the natural oxide HfO_X_, while the surface of Hf_50_B_20_ has a glassy phase that helps to insulate the oxygen, so it is difficult to form the natural oxide. Based on XPS spectroscopy, the ratio of HfO_2_ to HfB_2_ in Hf_50_B_20_ is about 3:2, indicating that HfB_2_ was mainly generated on the surface of ceramic particles.

Figure 13e,f show the full XPS spectra of Hf_50_B_0_ and Hf_50_B_20_ before and after ablation. The results surface that the elemental changes before and after ablation of Hf_50_B_0_ are not obvious. The intensity of the binding energy peaks associated with the N element in the XPS full spectrum was too low. This also indicates that there was little BN content in Hf_50_B_0_. However, the binding energy peaks associated with elemental N can be clearly observed in the XPS image of Hf_50_B_20_ after ablation, further confirming that BN is the main ablation product of Hf_50_B_20_.

### 3.6. Ablation Mechanisms

**Figure 14:**
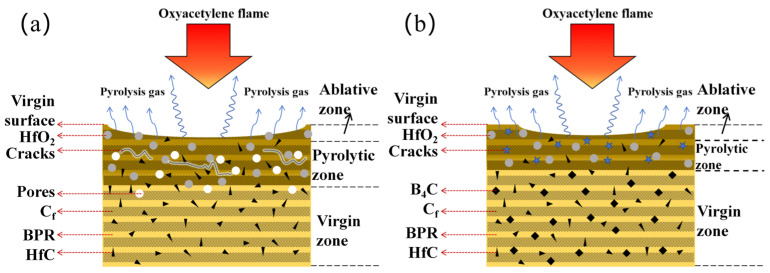
Schematic representation of the ablation mechanism of (**a**) Hf_50_B_0_ and (**b**) Hf_50_B_20_.

Based on the studies of the ablation behavior, microstructural evolution, and phase evolution, the possible ablation mechanisms were as follows:

For the Hf_50_B_0_ composite (Figure 14a), the pyrolysis of the BPR results in the detachment of discrete HfC and HfO_2_ particles, which were subsequently removed by high-speed airflow. This led to the exposure of a large number of carbon fibers on the ablation surface, along with an enlarged central ablation area.

After adding B_4_C, the ablation resistance of the composite material was improved. Taking Hf_50_B_20_ as an example (Figure 14b), the ablation surface was covered with a ceramic layer, so the area of carbon fibers exposed was reduced. The ceramic layer mainly consisted of ablation-resistant ceramics (HfC, HfO_2_, and HfB_2_ particles) as well as a continuous glass phase. The glassy phase composed mainly of B_2_O_3_ and BN exhibits fluidity, allowing it to fill and repair cracks and pores generated by the pyrolysis of the substrate. The ablation-resistant ceramics were adhered to or embedded in the glass phase.

In summary, a B–C–N–O–Hf multicomponent dense ceramic layer was constructed on the ablation surface of Hf_50_B_20_, forming an effective ablation-resistant barrier. The layer significantly impeded thermochemical erosion of the carbon fibers and PyC, while preventing thermomechanical stripping. Additionally, the strong heat absorption of HfO_2_, B_2_O_3_, and PyC during carbothermal reactions further enhanced the thermal protection performance of the material.

## 4. Conclusions

This report presents our study of the development and characterization of HfC–B_4_C-modified C_f_/BPR ceramizable composites, demonstrating their superior oxidative corrosion and ablation resistance. As the B_4_C content increased, the density of the composites increased, while the porosity decreased. The residual yield at 1400 °C first increased and then decreased. The highest residual yield of 46.0% was found for Hf_50_B_10_. There was no obvious pattern in the bending strength, but the bending strength of the samples with added B_4_C was significantly higher than that of the unadded ones. The flexural strength of Hf_50_B_20_ was as high as 54.65 MPa after heat treatment at 1600 °C for 20 min. A dense multicomponent ceramic layer was formed on the surface of the material after heat treatment. In the ablation test, both LAR and MAR first increased and then decreased with the increase in the B_4_C content. At a heat flux density of 4.2 MW/m^2^ for 20 s, the LAR of Hf_50_B_20_ was as low as −8.33 × 10^−3^ mm/s, and the MAR was 3.08 × 10^−2^ g/s. In summary, Hf_50_B_20_ has the best overall thermal protection performance.

The ablation mechanism was further revealed. Due to the synergistic effect of HfC and B_4_C, a dense B–C–N–O–Hf ceramic layer was formed in situ on the ablated surface. As an effective thermal protection barrier, this ceramic layer significantly prevents the thermochemical erosion of carbon fibers and PyC and prevents stripping by high-speed airflow. The carbothermal reaction between HfO_2_, B_2_O_3_, and PyC contributes to the thermal insulation capability of the composite due to its strong heat-absorption effect. These findings confirm the potential of HfC–B_4_C-modified C_f_/BPR ceramizable composites for applications in thermal protection systems.

## Figures and Tables

**Table 1:** Formulations of ceramizable composites.

Samples	Mass/g			
	BPR	C_f_	HfC	B_4_C
Hf_0_B_0_	100	100	0	0
Hf_50_B_0_	100	100	50	0
Hf_50_B_5_	100	100	50	5
Hf_50_B_10_	100	100	50	10
Hf_50_B_15_	100	100	50	15
Hf_50_B_20_	100	100	50	20

## Data Availability

The original contributions presented in this study are included in the article/Supplementary Material. Further inquiries can be directed to the corresponding authors.

## Supplementary Materials

The following supporting information can be downloaded at: https://www.mdpi.com/article/10.3390/polym17101412/s1. Figure S1: The flexural strength for similar studies in the literature; Figure S2: The ablation rates for similar studies in the literature; Figure S3: Cell images of (a) HfC, (b) m-HfO_2_, (c) c-HfO_2_ and (d) HfB_2_; Figure S4: Raman spectra of Hf_50_B_X_ surface phases after ablation; Table S1: The thermal conductivity and standard deviation of composites; Table S2: The specific values of flexural strength for similar studies in the literature; Table S3: The specific values for ablation rates for similar studies in the literature; Table S4: Cellular parameters of HfC, m-HfO_2_, c-HfO_2_ and HfB_2_.

## Abbreviations

The following abbreviations are used in this manuscript:

Cf	Carbon fiber
Ph	Phenolic resin
HfC	Hafnium carbide
B4C	Boron carbide
LAR	Linear ablation rate
MAR	Mass ablation rate
TPS	Thermal protection system
UHTCs	Ultra-high-temperature ceramics
BPR	Boron phenolic resin
SEM	Scanning electron microscope
EDS	Energy-dispersive X-ray
XRD	X-ray diffraction
XPS	X-ray photoelectron spectrometer
TGA	Thermogravimetric analysis
DTG	Derivative thermogravimetry
PyC	Pyrolytic carbon
ΔG	Gibbs free energy change
ΔH	Enthalpy change
