# Supplementary material for: Oxidation Resistance, Ablation Resistance, and Ablation Mechanism of HfC–B4C-Modified Carbon Fiber/Boron Phenolic Resin Ceramizable Composites
Source: Polymers (Basel). 2025 May 20;17(10):1412. doi: 10.3390/polym17101412 (PMC12114954; doi:10.3390/polym17101412)
Supplement: Supplementary file 1 [file polymers-17-01412-s001.zip › polymers-3616559-supplementary.docx]

**Supplementary Materials:**

| **Samples** | **Thermal conductivity**  **(W/(m·K))** | **Standard deviation**  **(σ)** |
| --- | --- | --- |
| Hf_0_B_0_ | 0.874 | 0.013 |
| Hf_50_B_0_ | 1.066 | 0.002 |
| Hf_50_B_5_ | 1.041 | 0.057 |
| Hf_50_B_10_ | 1.059 | 0.021 |
| Hf_50_B_15_ | 1.042 | 0.033 |
| Hf_50_B_20_ | 1.110 | 0.002 |

**Table S1.** The thermal conductivity and standard deviation of composites

**Table S2**


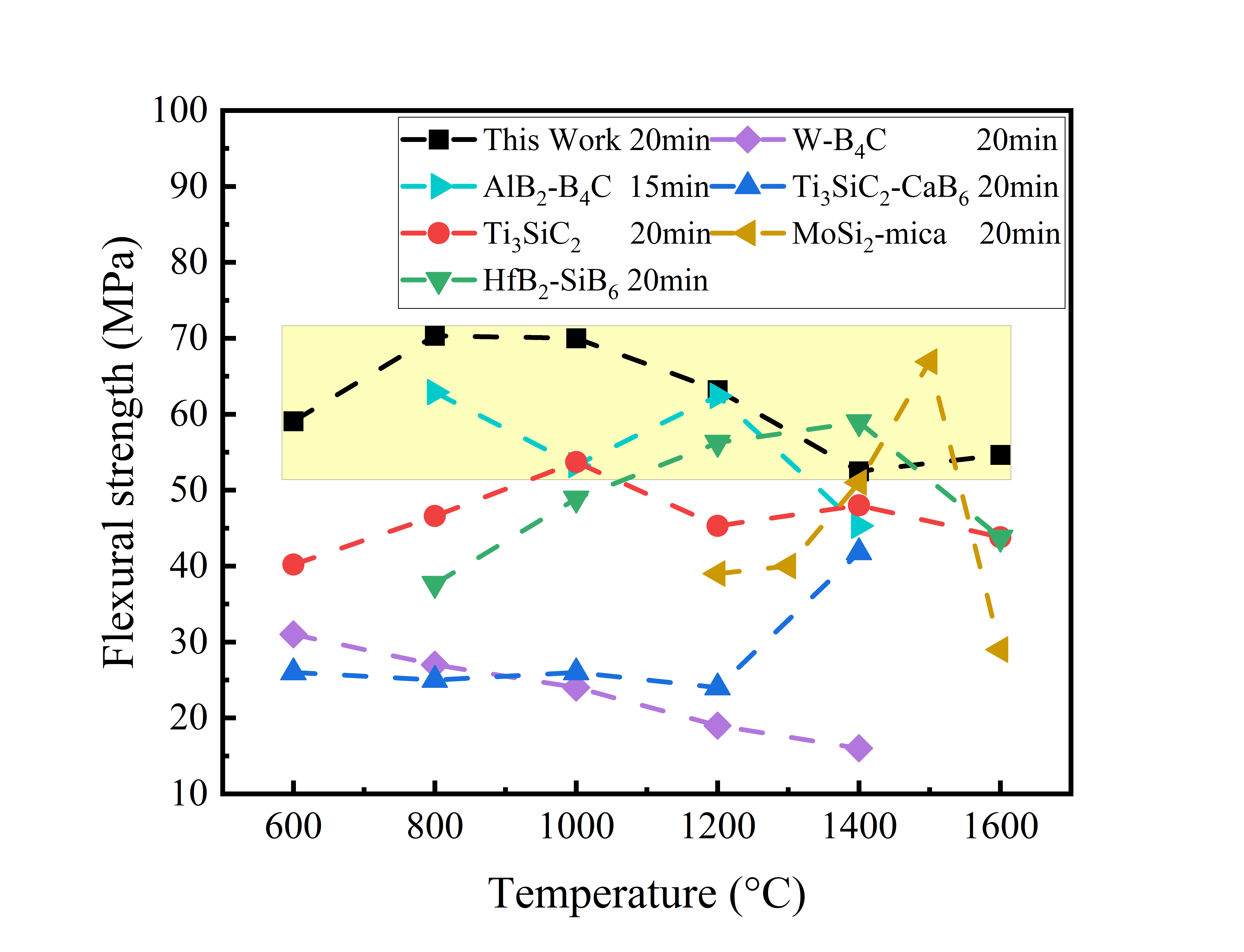


**Figure S1.** The flexural strength for similar studies in the literature

**Table S2.** The specific values of flexural strength for similar studies in the literature

| **Similar studies** | **Heat treatment time (min)** | **Flexural strength (MPa)** | | | | | | | |
| --- | --- | --- | --- | --- | --- | --- | --- | --- | --- |
|  |  | **600 °C** | **800 °C** | **1000 °C** | **1200 °C** | **1300 °C** | **1400 °C** | **1500 °C** | **1600 °C** |
| Ti_3_SiC_2_ | 20 | 40.2 | 46.6 | 53.7 | 45.3 | — | 48.0 | — | 43.8 |
| Ti_3_SiC_2_ - CaB_6_ | 20 | 26.1 | 25.4 | 26.7 | 24.6 | — | 41.8 | — | — |
| W - B_4_C | 20 | 31.0 | 27.2 | 24.1 | 19.9 | — | 16.3 | — | — |
| AlB_2_ - B_4_C | 15 | — | 62.9 | 53.3 | 62.4 | — | 45.3 | — | — |
| HfB_2_ - SiB_6_ | 20 | — | 37.7 | 48.9 | 56.2 | — | 59.0 | — | 43.7 |
| MoSi_2_ - mica | 20 | — | — | — | 38.9 | 40.4 | 51.2 | 66.9 | 29.0 |
| This Work | 20 | 59.1 | 70.3 | 70.0 | 63.2 | — | 52.5 | — | 54.7 |


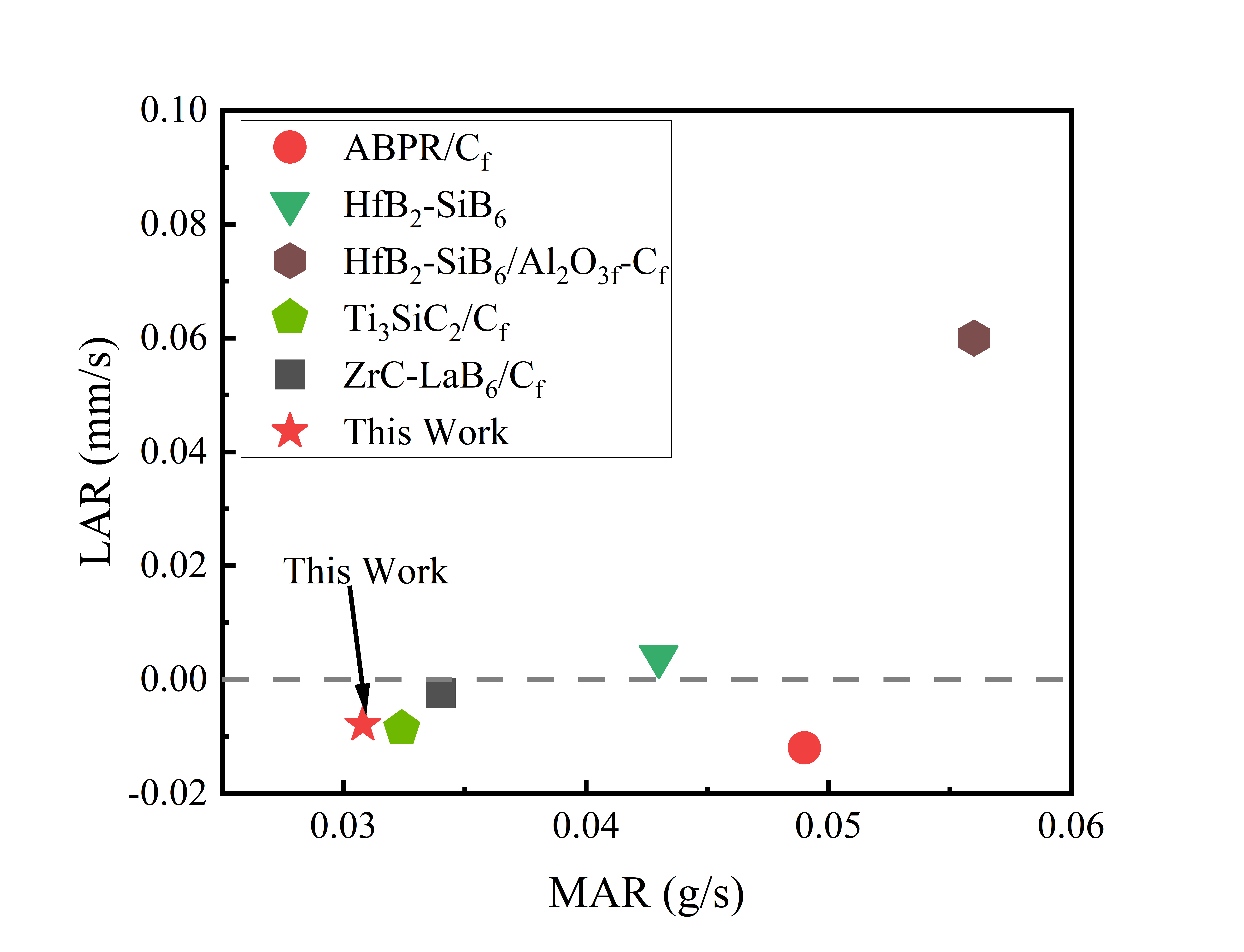


**Figure S2.** The ablation rates for similar studies in the literature

**Table S3.** The specific values for ablation rates for similar studies in the literature

| **The similar studies** | **The heat flow density (MW/m^2^)** | **Time (s)** | **LAR (**× 10^-3^ **mm/s)** | **MAR (**× 10^-2^ **g/s)** |
| --- | --- | --- | --- | --- |
| ABPR/C_f_ | 4.2 | 30 | -12 | 4.9 |
| HfB_2_ - SiB_6_ | 4.2 | 20 | 4 | 4.3 |
| HfB_2_ - SiB_6_/Al_2_O_3f_ - C_f_ | 4.2 | 20 | 60 | 5.6 |
| Ti_3_SiC_2_/C_f_ | 4.3 | 30 | -8.59 | 3.24 |
| ZrC - LaB_6_/C_f_ | 4.3 | 30 | -2.31 | 3.4 |
| This Work | 4.2 | 20 | -7.91 | 3.08 |

**Table S4.** Cellular parameters of HfC, m-HfO_2_, c-HfO_2_ and HfB_2_

| **Crystallography** | **a (**Å**)** | **b (**Å**)** | **c (**Å**)** | **α (**°**)** | **β (**°**)** | **γ (**°**)** |
| --- | --- | --- | --- | --- | --- | --- |
| HfC | 4.638 | 4.638 | 4.638 | 90.00 | 90.00 | 90.00 |
| m-HfO_2_ | 5.119 | 5.179 | 5.296 | 90.00 | 99.30 | 90.00 |
| c-HfO_2_ | 5.110 | 5.110 | 5.110 | 90.00 | 90.00 | 90.00 |
| HfB_2_ | 3.142 | 3.142 | 3.480 | 90.00 | 90.00 | 120.0 |


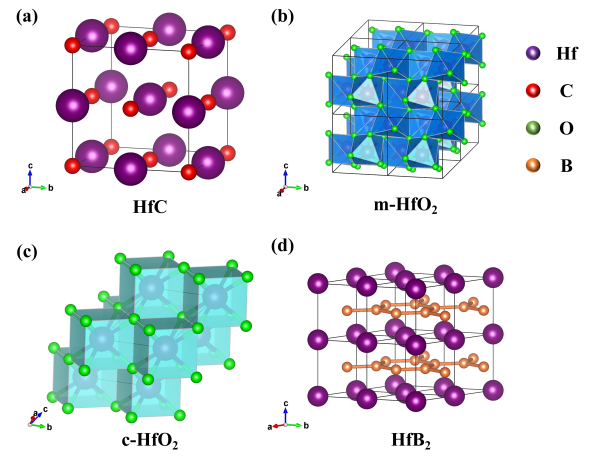


**Figure S3.** Cell images of (a) HfC, (b) m-HfO_2_, (c) c-HfO_2_ and (d) HfB_2_


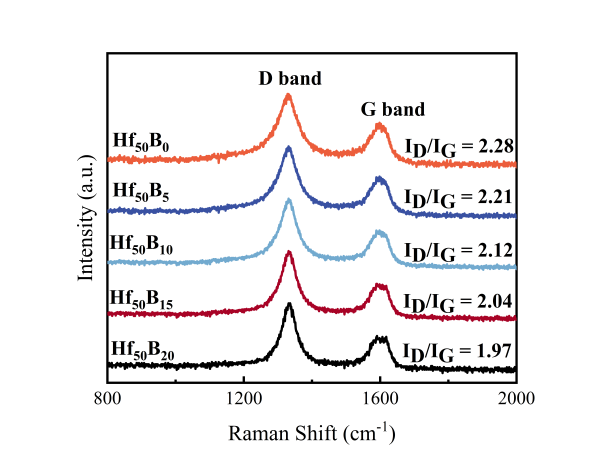


**Figure S4.** Raman spectra of Hf_50_B_X_ surface phases after ablation
